# Polyalcohols as Hydrogen-Bonding Donors in Choline Chloride-Based Deep Eutectic Solvents for Extraction of Xanthones from the Pericarp of *Garcinia mangostana* L.

**DOI:** 10.3390/molecules24030636

**Published:** 2019-02-12

**Authors:** Kamarza Mulia, Farah Fauzia, Elsa Anisa Krisanti

**Affiliations:** Department of Chemical Engineering, Universitas Indonesia, Depok 16424, Indonesia; farfazia@gmail.com (F.F.); elsakm@che.ui.ac.id (E.A.K.)

**Keywords:** deep eutectic solvent, solvent extraction, *Garcinia mangostana* L., mangosteen, α-mangostin, choline chloride, polyalcohol

## Abstract

Mangosteen (*Garcinia mangostana* L.) is a fruit that is rich in xanthones, utilized as health supplements or additives in food products due to their high antioxidant activities. Choline chloride (ChCl)-based deep eutectic solvents (DESs) with polyalcohols (ethylene glycol, glycerol, propanediols, and butanediols) as hydrogen bonding donors (HBDs) were used to extract the xanthones from the pericarp of mangosteen. DESs with 1,2-propanediol, 1,3-propanediol, and 1,2-butanediol as HBDs (ChCl to HBD mole ratio of 1:3) afforded the highest extraction yields (2.40-2.63%) of α-mangostin, the most abundant component that represents xanthones. These DESs have intermediate Nile Red polar parameter values similar to that of ethanol and provide extraction yields with a quadratic dependence on the polar parameter. Polarity and viscosity, the important physicochemical properties to consider in the selection of DES as an extraction solvent, could be adjusted based on the consideration of the molecular structure of the polyalcohols. The following factors qualifies the ChCl-1,2-propanediol DES as a designer solvent for green extraction: It is selected from a set of DESs having a homologous class of HBDs to deliver the highest α-mangostin extraction yield, its extract composition similar to that obtained using ethanol, it has low or negligible vapor pressure, both of its components are generally recognized as safe chemicals so that direct utilization of a DES extract is possible, and this DES is used for utilization of agricultural waste products as the resource of bioactive compounds.

## 1. Introduction

Mangosteen is a tropical fruit tree that grows in Southeast Asia. The fruit is rich in water, protein, carbohydrates, fiber, essential nutrients and many kinds of vitamins [1]. Phytochemical studies have shown that the mangosteen pericarp, which is a biomass waste, contains secondary metabolites, e.g., oxygenated and prenylated xanthones [2]. Xanthones are bioactive compounds that have antioxidant, antitumor, anti-inflammatory, antiallergy, antibacterial, antifungal and antiviral activities [3,4,5]. Most of the xanthones are found in the pericarp or rind, in an amount sixteen times higher than that in the aril segments [6]. The most abundant xanthone in the mangosteen pericarp is α-mangostin, while other xanthones are present in less amounts (γ-mangostin, β-mangostin, gartanin, and 8-deoxygartanin) [7]. Polyphenol substances, such as xanthones, extracted from plant or agriculture waste product, have been utilized as antioxidant food supplements. Recently xanthones in the form of mangosteen pericarp powder have been added to dark and compound chocolates [8].

Extraction of bioactive compounds from natural resources usually involves the use of a large amount of organic solvents. Most of these organic solvents possess a certain degree of toxicity and volatility, generating waste that is harmful to both human health and the environment. Due to their nonvolatility at ambient conditions compared with the volatile organic solvents, ionic liquids (ILs) have received significant attention as alternative solvents [9,10]. ILs are used in a wide range of applications [11,12], however, they are not commonly used in pharmaceutical and food industries due to their toxicity and high synthesis cost of some ILs [13,14,15].

The search of green solvents as substitutes for hazardous organic solvents in order to minimize environmental problems, improve safety and health, as well as reduce cost, has led to the use of deep eutectic solvents (DESs). They are a mixture of a hydrogen bond acceptor (HBA) molecule that forms intermolecular hydrogen bonds with one or more hydrogen bond donor (HBD) molecules, thereby decreasing the melting point of the mixture to temperatures much lower than those of its individual components. Abbott et al. were the first to report that DESs could be formed between choline chloride (ChCl), using a quaternary ammonium salt as a HBA, and a range of amides, carboxylic acids and alcohols as HBDs [16]. While DESs have physical properties similar to those of ILs [9,16,17], they are considered as alternatives to ILs due to their low vapor-pressure, ease of synthesis, biocompatibility, biodegradability, and low production cost [18,19]. Dai et al. [13] suggested the term Natural Deep Eutectic Solvents (NADES) for DESs that are mixtures of various cellular constituents or primary metabolites found in many kinds of organisms. NADES that are more stable as liquids at room temperature, having nontoxic and environmentally friendly characteristics, are potentially useful as solvents for extraction of bioactive compounds from plants [14].

In this study, extraction yields were measured in terms of α-mangostin, the most abundant xanthone in the mangosteen pericarp. A high extraction yield of α-mangostin extracted from the pericarp of mangosteen could be expected when the hydrogen bonding interactions in the ChCl-α-mangostin complex was similar in nature and strength to the interactions in the ChCl-polyalcohol complex [20,21]. Quantum mechanical and molecular dynamics simulations of the ChCl-urea, ChCl-ethylene glycol, and ChCl-glycerol DESs showed that the melting point depression and viscosity data of these DESs could be correlated directly to the strength and nature of the hydrogen bond network in the bulk liquid [22].

Previously, we reported that ChCl-based DESs with 1,2-propanediol (a polyalcohol) as the HBD gave α-mangostin extraction yields in the range of 2.0–2.5%, much higher than the yields obtained using DESs with citric acid, glycerol, and glucose as the HBDs (<0.5%) [23]. This finding deserves further investigation of a homologous class of polyalcohols used as HBDs in ChCl-based DESs, regarding their effectiveness as extracting solvents for xanthones. It is anticipated that this approach would allow a systematic investigation of the effect of the molecular structure of the polyalcohols, and the physicochemical properties of the DESs, on the extraction yields. It would be of interest to see if a DES could provide a high extraction yield, and also, exemplify a designer solvent for a green extraction.

In this study, we tested 17 DESs, each consisting of ChCl and a polyalcohol (ethylene glycol, glycerol, 1,2-propanediol, 1,3-propanediol, 1,2-butanediol, 1,3-butanediol, 1,4-butanediol) as the HBD. Our first objective was to investigate how extraction yields of α-mangostin was affected by the composition of the DESs, the molecular structure of the HBDs, and the physicochemical properties of the DESs. The second objective was to determine if ChCl-polyalcohol DESs was a feasible substitute for ethanol, a common organic solvent used for the extraction of bioactive compounds from plants.

## 2. Results and Discussion

### 2.1. Density, Polarity and Viscosity of ChCl-Polyalcohol DESs

Table 1 gives the density, polarity and viscosity data of the ChCl-polyalcohol DESs. The density data obtained was used to calculate extraction yields, while the polarity and viscosity data was measured to see the effect of these physicochemical properties on the mangostin extraction yields. DESs were prepared by mixing choline chloride as the HBA and a polyalcohol as the HBD, in three mole ratios (1:1, 1:2, and 1:3), until a homogenous solution was obtained. Mixtures that were unstable or took too long to form were not included in this study.

The polarities of the DESs were determined in terms of their Nile Red polar parameter (E_NR_) values, calculated using Equation (1), where a lower E_NR_ value indicated a more polar solvent [24]. Accordingly, the polarity of the DESs in descending order was as follows: DES-EG (ethylene glycol) > DES-2B (1,2-butanediol) ~ DES-3B (1,3-butanediol) > DES-G1 (glycerol) > DES-2P (1,2-propanediol) ~ DES-3P (1,3-propanediol) > DES-4B (1,4-butanediol).

The viscosity of all DESs decreased with increasing polyalcohol to ChCl mole ratio, due to the increased solvation effect as more hydroxyl groups were available around the chloride anion of the ChCl molecule. Reduced viscosity was also observed for DESs with polyalcohols having shorter methylene chains between the two hydroxyl groups, in the order of DES-EG (ethylene glycol) < DES-3P (1,3-propanediol) < DES-4B (1,4-butanediol). The position of the OH groups in a polyalcohol also affected the viscosity of a DES as DES-2B (1,2-butanediol) and DES-3B (1,3-butanediol) had the lowest and highest viscosities, respectively.

### 2.2. Optimum Extraction Time

**Figure 1:**
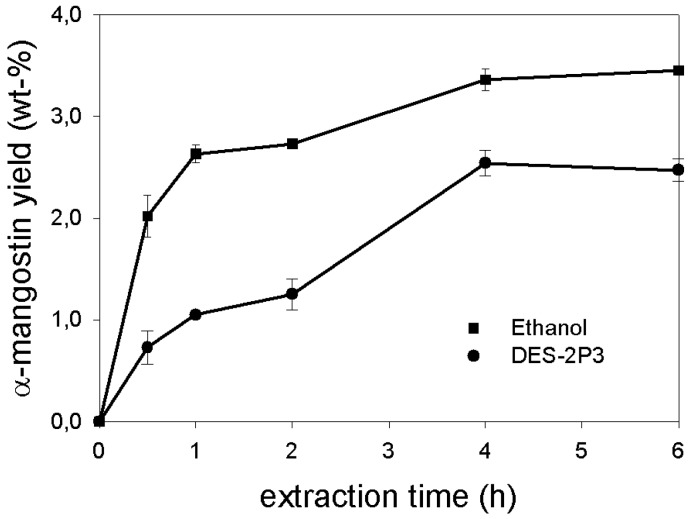
Plots of α-mangostin yield as a function of extraction time. Data obtained using choline chloride-1,2-propanediol deep eutectic solvent (DES) (mole ratio of 1:3) and ethanol as extracting solvents.

Plots of the extraction yield data as a function of extraction time, obtained using ethanol and DES-2P3 as the extracting solvents, are shown in Figure 1. The much lower initial extraction rate of the DES, compared to that of ethanol, could be explained using the Stokes-Einstein equation. The diffusivity of DES-2P3 was lower than that of ethanol due to its higher viscosity and the larger size of the ChCl-1,2-propanediol complex, making it more difficult for this solvent to infiltrate the pericarp matrix. The two plots levelled off after 4 h, an indication that the equilibrium condition had been attained. Accordingly, an extraction time of 4 h was used in the subsequent experiments.

### 2.3. α-Mangostin Extraction Yields

**Figure 2:**
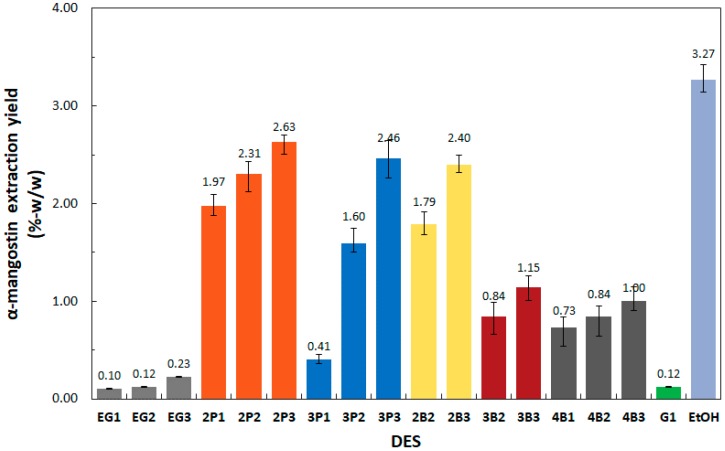
Extraction yield obtained using ChCl-polyalcohol DESs. The error bar indicates the maximum and minimum yields (*n* = 3). Label: EG (ethylene glycol), 2P (1,2-propanediol), 3P (1,3-propanediol), 2B (1,2-butanediol), 3B (1,3-butanediol), 4B (1,4-butanediol), G (glycerol), EtOH (ethanol), last digit (mole ratio of polyalcohol to ChCl).

α-mangostin extraction yield data obtained using 17 ChCl-based DESs, defined as the mass percentage of α-mangostin extracted from the dried mangosteen powder, is given in Table 1 and shown in Figure 2. The effect of the choline chloride to polyalcohol mole ratio, molecular structure of the polyalcohols, and polarity and viscosity of the DESs are discussed in turn.

#### 2.3.1. Effect of ChCl to Polyalcohol Mole Ratio

The effect of ChCl to polyalcohol mole ratio on extraction yield was exhibited most clearly by DES-3P1 and DES-3P3 where sixfold improvement in yield (0.41 to 2.46%) was obtained by increasing ChCl to 1,3-propanediol mole ratio from 1:1 to 1:3, respectively. This effect was due to the presence of more hydroxyl groups of the polyalcohol, surrounding the negatively charged chloride anion of ChCl, decreasing the polarity of the DESs, and inducing more dissolution of the non-polar α-mangostin molecules. The slightly basic choline chloride in DESs increased the extraction efficiency due to the weak acidity of the phenolic OH groups of α-mangostin [25].

#### 2.3.2. Effect of Molecular Structure of Polyalcohols

**Figure 3:**
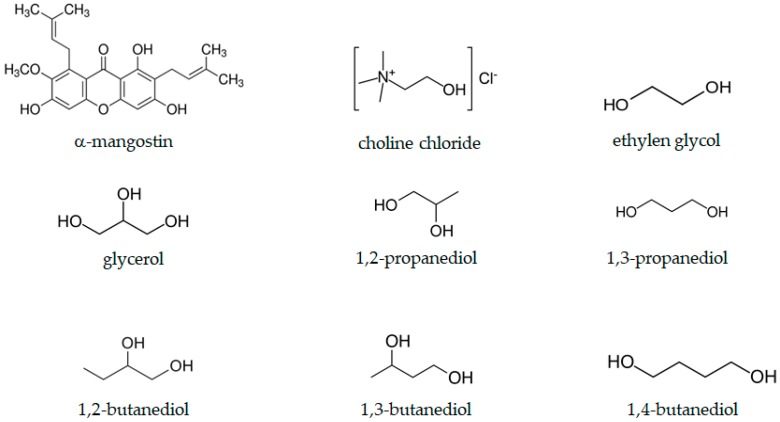
Chemical structure of α-mangostin, choline chloride, and polyalcohols used in this study.

Choline chloride was the only hydrogen-bonding acceptor used in this study, therefore, the extraction yield data could be analyzed systematically based on the molecular structure of the polyalcohols (Figure 3) used as the hydrogen-bonding donor. The highest extraction yields were obtained using DES-2P3 (2.63%) and DES-2B3 (2.40%), having HBDs with adjacent OH groups in a terminal position attached to a methyl group (1,2-propanediol) and an ethyl group (1,2-butanediol), respectively. The alkyl chain allowed the more polar OH groups of α-mangostin to draw closer to the chloride anion of ChCl due to hydrogen bonding. This meant that the alkane chain would be directed away from the ChCl, increasing the void volume of the DESs necessary for extraction of α-mangostin. This explanation was consistent with the very low extraction yield of 0.23% observed for DES-EG3 (ethylene glycol). Without any methylene group, the more polar ethylene glycol seemed to form a more dense complex with choline chloride [21], making DES-EG3 not conducive to the solubilization of a large and slightly non-polar molecule such as α-mangostin.

The length of the straight carbon chain between two hydroxyl end groups in HBDs tested was (in decreasing order): 1,4-butanediol (DES-4B3) > 1,3-propanediol (DES-3P3) > ethylene glycol (DES-EG3). DES-3P3, having 1,3-propanediol with one methylene group between the two hydroxyl end groups, was able to extract a higher amount of α-mangostin (2.46%) than DES-4B3 having 1,4-butanediol with two methylene groups (1.00%) or DES-EG3 having ethylene glycol with no methylene group (0.23%). This observation suggested that the molecular structure of 1,3-propanediol is spatially preferable to form a hydrogen-bonded complex with ChCl, with interactions similar to those between choline chloride and α-mangostin. A similar study regarding the molecular structure of 1,4-butanediol and possibility of forming hydrogen bonding with choline chloride has been reported earlier [21].

The position of the OH groups along the alkane chain of the butanediol isomers also has a significant effect on the extraction yields. The yields for DES-2B3 (1,2-butanediol), DES-3B3 (1,3-butanediol), and DES-4B3 (1,4-butanediol) were 2.40%, 1.15%, and 1.00%, respectively. As discussed previously, the alkane chain in 1,2-butanediol was the most flexible, allowing the adjacent terminal OH groups to be attracted to the charged part of choline chloride more easily.

#### 2.3.3. Effect of Polarity of DESs

**Figure 4:**
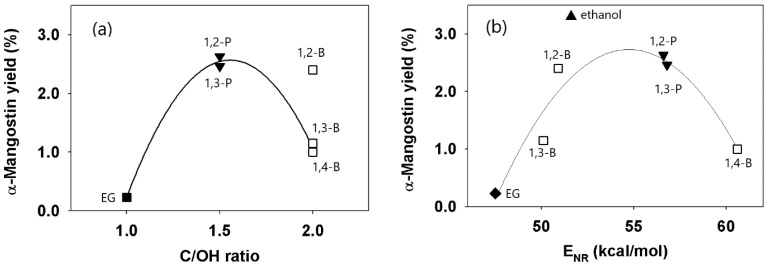
Extraction yield as a function of: (**a**) the C/OH ratio of the polyalcohols, and, (**b**) polar parameter E_NR_ of the DESs. (ChCl:polyalcohol mole ratio of 1:3, legend signifies the HBD of the DES).

To study the effect of polarity of a DES on extraction of α-mangostin, extraction yield data of DESs with a ChCl to polyalcohol mole ratio of 1:3 was plotted against the carbon to hydroxyl (C/OH) ratio of the HBDs. The extraction yield data of DESs with ChCl to polyalcohol mole ratios of 1:1 and 1:2 were not included in the plot as they did not reveal a systematic pattern as was in the case of DESs with the 1:3 ratio. Figure 4a shows that the highest extraction yields were obtained using DESs of an HBD with a C/OH ratio of 1.5, while significantly lower yields were obtained using DESs that were not sufficiently polar or too polar, having HBDs with C/OH ratios of 2 and 1, respectively. An approximately quadratic plot was obtained, excluding the data of DES-2B3.

To obtain a better extraction yield dependence on polarity, the Nile Red polar parameter (E_NR_) was used to represent the polarity of the DESs. The resulting plot (Figure 4b) shows that the α-mangostin yield had an approximately quadratic dependence on the E_NR_ value of the DESs (R^2^ of 0.91, excluding data of ethanol), and DESs with high extraction yields had intermediate E_NR_ values (50.6–56.8 kcal/mol) that bounded the E_NR_ value of ethanol (51.6 kcal/mol). The plot could be useful in the absence of prior experimental data, for example, DES-2B3 (50.9 kcal/mol) and not DES-4B3 (60.6 kcal/mol), could be selected as an extraction solvent based on the similarity to the E_NR_ value of ethanol (51.6 kcal/mol).

The highest extraction yield of 3.3% was obtained using ethanol, slightly higher than 2.63% yield obtained using DES-2P3. The difference is almost statistically insignificant (t_calc_ = 4.34, t_table_ = 4.30, α = 0.05, *n* = 3). The data obtained in this study showed that three ChCl-polyalcohol DESs were effective extraction solvents and they could be used as substitutes for ethanol to extract bioactive compounds from plants. Very low extraction yields were obtained using DESs with relatively more polar HBDs, ethylene glycol (DES-EG3) and glycerol (DES-G1), both having a C/OH ratio of 1. These results suggest that DES polarity was an important factor to consider in order to obtain high extraction yields of bioactive compounds such as α-mangostin.

#### 2.3.4. Effect of Viscosity of DESs

For a certain DES, the effect of viscosity predominated over the effect of solvent polarity. As shown in Figure 4a,b, the extraction yield of DES-2B3 was much higher (2.40%) than that of DES-3B3 (1.15%) and DES-4B3 (1.00%), in spite of the fact that both 1,2-butanediol and 1,3-butanediol had the same C/OH ratio of 2 and very similar E_NR_ values. In the case of DES-2B3, both the viscosity and the polarity exerted a synergistic effect on extraction yield. On the other hand, the much lower yield of DES-3B3 seemed to be due to its high viscosity value, in contrast to DES-2B3, which had the lowest viscosity among all of the DESs tested.

### 2.4. Composition of Xanthones in Ethanolic and DES Extracts

**Figure 5:**
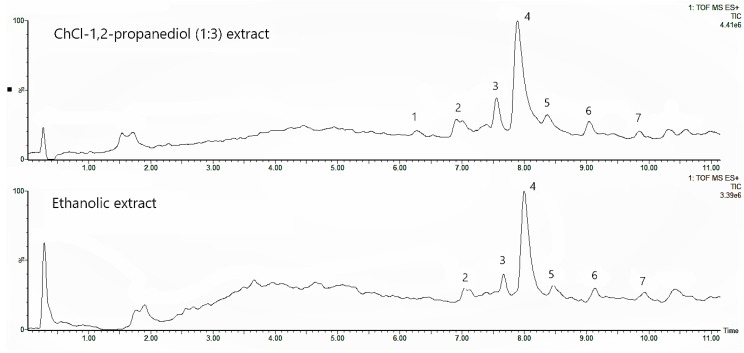
Chromatogram of the DES-2P3 (above) and ethanolic extracts (below). Numbers indicate the xanthone components identified using LC-MS: (**1**) 1,7-dihydroxy-3-methoxy-2-(3-methylbut-2- enyl)xanthon, (**2**) γ-mangostin, (**3**) gartanin, (**4**) α-mangostin, (**5**) garcinone E, **(6**) garcimangosone B, (**7**) β-mangostin.

The xanthones recovered from the DES that gave the highest extraction yield (DES-2P3) were identified using an LC-MS analyzer. The relative compositions of xanthones in DES and in ethanol, were determined based on the area percentage of each specific peak compared with the overall area of the major peaks (Figure 5).

Table 2 provides the composition of the DES extract that was dominated by α-mangostin (α-mangostin, γ-mangostin, and a small percentage of β-mangostin) followed by gartanin, garcinone E, and garcimangosone B, present in less amounts. Even though one of the compounds present in DES extract, 1,7-dihydroxy-3-methoxy-2-(3-methylbut-2-enyl)xanthon, was not detected in the ethanolic extract, it was detected as a minor compound in another similar study [6]. The result of the LC-MS analysis showed that DES and ethanol had similar capabilities in extracting xanthones.

The similarity of both the α -mangostin extraction yields and the composition of the extracted xanthones indicated that DES-2P3 could be used to extract bioactive compounds, as a substitute for ethanol, the commonly used organic solvent. Around 39.9% of the α -mangostin in DES-2P3 was recovered in a single step using ethyl acetate and diethyl ether as the back-extracting solvents, therefore, multiple back-extraction was required to improve the recovery [18].

### 2.5. ChCl-1,2-Propanediol DES as a Designer Solvent for Green Extraction

DESs are known as designer solvents that allow for a systematic search for the optimum HBA-HBD combination and mole ratio, suitable for the extraction of the targeted bioactive compounds. In this study, a set of DESs having a homologous class of polyalcohols was screened for the best solvent for the extraction of α-mangostin. It was found that ChCl-based DESs having 1,2-propanediol, 1,3-propanediol and 1,2-butanediol as HBDs (in a mole ratio of 1:3) afforded high extraction yields of xanthones, in terms of α-mangostin. The ChCl-1,2-propanediol was the DES with the highest extraction yield of α-mangostin among the seventeen polyalcohol-DESs tested, presumably, by forming choline chloride and a 1,2-propanediol complex with sufficient space, polarity, and viscosity suitable for effective interaction with α-mangostin. This DES exemplified the advantages of DESs as designer solvents by combining high extraction yield, low or negligible vapor pressures, and good biodegradability aspects [26,27].

There are two additional advantages of using the ChCl-1,2-propanediol DES. Firstly, choline chloride and 1,2-propanediol are categorized as generally recognized as safe (GRAS) chemicals [28]. The use of biocompatible HBAs and HBDs opens up the possibility of direct utilization of DES extracts as part of the bioactive compound formulation, eliminating the back-extraction and solvent recovery steps. Secondly, the xanthones were extracted from the pericarp of mangosteen, an agricultural waste product. These advantages are in line with the principles of green extraction proposed by Chemat et al. [29]. When taken together, the results of this study qualifies the ChCl-1,2-propanediol DES as a designer solvent for green extraction.

## 3. Materials and Methods

### 3.1. Chemicals and Plant Material

Choline chloride (>98%), 1,2-propanediol (99%), 1,3-propanediol (99%), 1,2-butanediol (99%), 1,3-butanediol (99%), 1,4-butanediol (99%), ethylene glycol (99%), glycerol (99%), and analytical grade Nile Red were purchased from Sigma Aldrich (Singapore). HPLC grade acetonitrile and ethanol were purchased from Smart Lab (Jakarta, Indonesia). The α-mangostin standard (>99.8%) was purchased from Aktin Chemical Inc., (Chengdu, China). Mangosteen fruit was purchased in Depok (West Java, Indonesia) and identified as *G. mangostana* by the Bogoriense Herbarium, Research Center for Biology, Indonesian Institute of Sciences, specimen voucher number 248/IPH.1.02/If.8/II/2014.

### 3.2. Preparation of Mangosteen Powder and ChCl-Polyalcohol DESs

Mangosteen pericarp was cleaned from its edible parts, the tough outer skin was peeled, inner part cut and dried at room temperature, and grind using an electric grinder (IKA, Staufen, Germany). The powder obtained was further dried in an oven at 65 °C until it reached constant weight, screened using a 20 mesh sieve, and stored in a sealed container to avoid contact with air and exposure to direct sunlight. DESs were prepared by mixing ChCl and polyalcohols in three mole ratios (1:1, 1:2, 1:3) under a constant stirring speed at a temperature of 50 and 80 °C, for liquid and solid polyalcohols, respectively. Stirring was continued for 30–90 min until a clear solution was formed [13]. Addition of water to DESs has been shown to be beneficial in reducing viscosity and increasing extraction yields [9], however, no water was added into the DESs used in this study to simplify the subsequent solvent recovery process as part of the overall extraction process.

### 3.3. Physicochemical Properties of DESs

The physicochemical properties determined in this study were density, polarity, and viscosity. Density measurement was carried out by weighing a known volume of DES dispensed from a micropipette previously calibrated using purified water. The Nile Red dye was added to each DES as a solvatochromic probe to determine λ_max_, the wavelength at which the maximum visible light absorption occurred [30]. A UV-vis spectrophotometer (Model UV-1900, Shimadzu, Kyoto, Japan) was used to scan the DES-dye mixtures in the 400–700 nm range and Equation (1) was used to calculate the Nile Red polar parameter E_NR_ (in kcal/mol). Viscosity test was performed using a Brookfield viscometer at room temperature with LV spindle no. 1 at 6, 12, and 30 rpm.

E_NR_ (kcal/mol) = 28,591/λ_abs,max_ (nm) (1)

### 3.4. Extraction of α-Mangostin Using Polyalcohol DESs and Ethanol

The extraction of α-mangostin was carried out by mixing 0.2 g of mangosteen powder and 2 g of DES or ethanol (solid to liquid mass ratio of 1:10) in a sealed reaction tube. The tube was shaken using a thermomixer apparatus (Model SCIENTZ-100, SCIENTZ, Ningbo, China) at a frequency of 500 oscillations/min at room temperature for 4 h. The suspension was then centrifuged for 15 min at 2000 rpm and the residue was separated using a 0.45 μm membrane filter to obtain the DES and ethanolic extracts. Each set of extraction was done in triplicate. The extraction time required for the process to reach equilibrium was determined based on the extraction yield data of a selected DES plotted against extraction time up to 6 h.

### 3.5. HPLC Analysis of α-Mangostin Extracted into DESs

The amounts of α-mangostin extracted into each DES formulation were determined using a high performance liquid chromatography (HPLC) analyzer (Model Prominence SPD-20A, Shimadzu, Kyoto, Japan), operated under conditions similar to those reported by [31]. The HPLC was equipped with an LC-20AD pump, a UV-Vis detector, a Rheodyne injector with 20 μL loops, and a C18 column with a length of 250 mm and a diameter of 4.6 mm. An isocratic elution mode was employed with a mobile phase flow rate of 1 mL/min, consisting of 0.1% (v/v) ortho-phosphoric acid solution and acetonitrile in equal volumetric flow rate. Sample injection volume was 0.1 mL and UV-Vis detector wavelength was set at 244 and 320 nm. Prior to injection, each sample was diluted with ethanol and filtered through a 0.45 μm membrane (Sartorius NY). The extraction yields were calculated as the percent ratio of the mass of the extracted α-mangostin to the mass of the dried mangosteen pericarp powder:

Mangostin yield (%) = 100 × mass of extracted α-mangostin/mass of dried mangosteen powder(2)

### 3.6. Recovery of Xanthones from DESs

DES with the highest α-mangostin extraction yield was chosen for the determination of α-mangostin recovery using the following procedure. First, 0.5 mL ethyl acetate was added to 1 mL mangostin-DES extract, stirred for 1 h, and decanted. An aliquot (0.35 mL) of the upper ethyl acetate fraction was separated for HPLC analysis. Then, 0.5 mL diethyl ether was added into the remaining DES mixture, stirred for another 1 h, and decanted. An aliquot (0.4 mL) of the upper fraction was separated for HPLC analysis. The total amount of α-mangostin recovered from the DES was the sum of α-mangostin extracted into the ethyl acetate and diethyl ether fractions.

### 3.7. LC-MS Analysis of α-Mangostin Extracted Using DES and Ethanol

The compositions of xanthones in the ethanol and the representative DES extract were determined using an LC-MS analyzer (Model ACQUITY UPLC^®^ H-Class System, Waters, Milford, MA, USA) equipped with a 1.7 µm pore size, 2.1 mm dia. × 50 mm BEH C18 column (Waters, Milford, MA, USA) and a photodiode array detector. The DES was mixed with a mixture of ethyl acetate and diethyl ether (volume ratio of 1:1), the mixture stirred and decanted, and the organic phase dried using a vacuum dryer. The dried mangostin extract was diluted using ethanol, filtered through a 0.2 µm membrane filter, and subjected to qualitative and quantitative analysis using the LC-MS analyzer. The sample was eluted employing a linear gradient mode over a 15 min period followed by an isocratic elution over a 2 min period, with a flow rate of 0.4 mL/min and the eluent composition of 95:5 to 5:95 (%-v/v) aqueous solution of formic acid (0.005%-v/v) and acetonitrile. The MS analysis was performed on a Xevo^®^ G2-XS QTof (Waters, Milford, MA, USA), employed in the positive ion mode and the electrospray ionization. The source temperature and the desolvation temperature were maintained at 120 and 500 °C, respectively. The desolvation gas flow was 844 L/h at a collision energy of 10 eV, and the ramp collision energy was 15–50 V. For comparative purposes, the ethanolic extract was analyzed without the pretreatment step.

## 4. Conclusions

The α-mangostin extraction yields were highly dependent on the polyalcohol and choline chloride to polyalcohol mole ratio of the DES used as the extracting solvent. The ChCl-based DESs that had 1,2-propanediol, 1,3-propanediol, and 1,2-butanediol as HBDs afforded high α-mangostin extraction yields, presumably by providing sufficient space and suitable polarity for an effective interaction between α-mangostin and the ChCl-polyalcohol complex. These DESs had intermediate Nile Red polar parameter values similar to that of ethanol, suggesting that polarity was an important factor to consider in the selection of a DES in order to obtain high extraction yields. In turn, polarity and viscosity, the most important physicochemical properties to consider in the selection of DES as an extraction solvent, could be adjusted based on the consideration of the molecular structure of the polyalcohols. The results of this study qualifies the ChCl-1,2-propanediol DES as a designer solvent for green extraction.

## Figures and Tables

**Table 1:** Compositions, physical properties, and extraction yields of choline chloride-based polyalcohol DESs.

Polyalcohol (HBD)	ChCl:HBD Mole Ratio	DES	Density (g/mL)	E_NR_ (kcal/mol) *	Viscosity (cP)	α-Mangostin Extraction Yield (%) **
Ethylene glycol	1:1	EG1	1.08	47.4	31.2	0.10 ± 0.01
	1:2	EG2	1.05	47.4	30.4	0.12 ± 0.01
	1:3	EG3	1.04	47.5	29.7	0.23 ± 0.01
1,2-Propanediol	1:1	2P1	1.05	56.5	37.1	1.97 ± 0.11
	1:2	2P2	1.03	56.7	35.2	2.31 ± 0.16
	1:3	2P3	1.01	56.6	31.6	2.63 ± 0.11
1,3-Propanediol	1:1	3P1	1.04	56.1	40.1	0.41 ± 0.05
	1:2	3P2	1.03	56.7	39.2	1.60 ± 0.13
	1:3	3P3	1.10	56.8	38.1	2.46 ± 0.19
1,2-Butanediol	1:2	2B2	1.06	50.6	27.6	1.79 ± 0.12
	1:3	2B3	1.06	50.9	25.8	2.40 ± 0.09
1,3-Butanediol	1:2	3B2	1.05	50.6	67.0	0.84 ± 0.17
	1:3	3B3	1.04	50.1	62.2	1.15 ± 0.13
1,4-Butanediol	1:1	4B1	1.10	57.8	44.8	0.73 ± 0.17
	1:2	4B2	1.05	58.8	43.9	0.84 ± 0.17
	1:3	4B3	1.05	60.6	43.7	1.00 ± 0.13
Glycerol	1:1	G1	1.14	55.6	46.8	0.12 ± 0.01
Ethanol	-	EtOH	-	51.6	-	3.27 ± 0.14

* Calculated using Equation (1); ** calculated using Equation (2) and reported as mean ± SD (*n* = 3).

**Table 2:** Composition of xanthones in DES-2P3 and ethanol based on the LC-MS data.

Compound	Xanthone	Formula	[M + H]^+^ m/z	Composition * (%)	
				Ethanol	DES
**1**	1,7-dihydroxy-3-methoxy-2-(3-methylbut-2-enyl)xanthon	C_19_H_18_O_5_	327	-	5.8
**2**	γ-mangostin	C_23_H_24_O_6_	397	13.0	12.8
**3**	gartanin	C_23_H_24_O_6_	397	16.0	12.3
**4**	α-mangostin	C_24_H_26_O_6_	411	53.6	52.4
**5**	garcinone E	C_28_H_32_O_6_	465	9.5	10.4
**6**	garcimangosone B	C_24_H_24_O_6_	409	4.7	4.2
**7**	β-mangostin	C_25_H_28_O_6_	425	3.3	2.1

* Composition estimates based on the normalized area of HPLC peaks.
